# Stereotactic Radiosurgery for Recurrent Meningioma: A Systematic Review of Risk Factors and Management Approaches

**DOI:** 10.3390/cancers17172750

**Published:** 2025-08-23

**Authors:** Yuka Mizutani, Yusuke S. Hori, Paul M. Harary, Fred C. Lam, Deyaaldeen Abu Reesh, Sara C. Emrich, Louisa Ustrzynski, Armine Tayag, David J. Park, Steven D. Chang

**Affiliations:** Department of Neurosurgery, Stanford University School of Medicine, Stanford, CA 94305, USA

**Keywords:** recurrent meningioma, stereotactic radiosurgery, radiation therapy, salvage treatment, Gamma Knife

## Abstract

*Background/Objectives*: Recurrent meningiomas remain difficult to manage due to the absence of effective systemic therapies and comparatively high treatment failure rates, particularly in high-grade tumors. Stereotactic radiosurgery (SRS) offers a minimally-invasive and precise option, particularly for tumors in surgically complex locations. However, the risks associated with re-irradiation, and recent changes in the WHO classification of CNS tumors highlight the need for more personalized and strategic treatment approaches. This systematic review evaluates the safety, efficacy, and clinical considerations for use of SRS for recurrent meningiomas. *Methods*: In accordance with the Preferred Reporting Items for Systematic Reviews and Meta-Analyses (PRISMA) guidelines, a systematic literature search was conducted using the PubMed, Scopus, and Web of Science databases for studies reporting outcomes of SRS in recurrent, pathologically confirmed intracranial meningiomas. Studies were excluded if they were commentaries, reviews, case reports with fewer than three cases, or had inaccessible full text. The quality and risk of bias of the included studies were assessed using the modified Newcastle-Ottawa Scale. Data on patient and tumor characteristics, SRS treatment parameters, clinical outcomes, adverse effects, and statistical analysis results were extracted. *Results*: Sixteen studies were included. For WHO Grade I tumors, 3- to 5-year progression-free survival (PFS) ranged from 85% to 100%. Grade II meningiomas demonstrated more variable outcomes, with 3-year PFS ranging from 23% to 100%. Grade III tumors had consistently poorer outcomes, with reported 1-year and 2-year PFS rates as low as 0% and 46%, respectively. SRS performed after surgery alone was associated with superior outcomes, with local control rates of 79% to 100% and 5-year PFS ranging from 40.4% to 91%. In contrast, tumors previously treated with radiotherapy, with or without surgery, showed substantially poorer outcomes, with 3- to 5-year PFS ranging from 26% to 41% and local control rates as low as 31%. Among patients with prior radiotherapy, outcomes were particularly poor in Grade II and III recurrent tumors. Toxicity rates ranged from 3.7% to 37%, and were generally higher for patients with prior radiation. Predictors of worse PFS included prior radiation, older age, and Grade III histology. *Conclusions*: SRS may represent a reasonable salvage option for carefully selected patients with recurrent meningioma, particularly following surgery alone. Outcomes were notably worse in high-grade recurrent meningiomas following prior radiotherapy, emphasizing the prognostic significance of both histological grade and treatment history. Notably, the lack of molecular and genetic data in most existing studies represents a key limitation in the current literature. Future prospective studies incorporating molecular profiling may improve risk stratification and support more personalized treatment strategies.

## Simple Summary

Management of recurrent meningiomas remains challenging, with limited evidence to support the use of systemic therapies. As a result, resection, radiotherapy, and stereotactic radiosurgery (SRS) remain the main therapeutic strategies. Among these, SRS has demonstrated promise as a minimally invasive treatment modality for select patients with recurrent meningiomas, offering favorable tumor control rates while minimizing risks to surrounding structures. This systematic review explores key factors that influence SRS treatment outcomes in this patient population, including tumor characteristics, prior therapies, and radiation dose parameters. We additionally consider the safety profile of SRS and highlight emerging strategies to reduce treatment-related risks through individualized planning.

## 1. Introduction

Meningiomas are among the most common primary brain tumors in adults, accounting for 20 to 35% of all such malignancies [1,2,3]. The World Health Organization classification of central nervous system (CNS) tumors categorizes meningiomas into three malignancy grades, which inform prognostication and clinical management of these lesions [4]. Approximately 80% of meningiomas are benign lesions (WHO Grade I) and have traditionally been managed with gross total resection (GTR) [5]. However, recurrence remains a significant concern, even following successful GTR [6]. High-grade meningiomas, namely WHO Grade II (atypical) and III (malignant or anaplastic) tumors, are associated with significantly higher recurrence rates, aggressive behavior, and poor survival outcomes [4,7]. Furthermore, high-grade meningiomas frequently exhibit resistance to multidisciplinary treatment approaches, therefore posing a significant therapeutic challenge [6,8,9]. While repeat resection may be required in some cases, this is associated with increased complexity and heightened risks of surgical complications [10,11].

With the advent of stereotactic radiosurgery (SRS), a minimally invasive treatment modality, the therapeutic landscape for meningiomas has significantly evolved [12,13]. It delivers focused, high-dose radiation to tumors while sparing surrounding healthy tissue, offering excellent local tumor control rates and minimal complications [8]. This is particularly valuable for tumors located near critical structures where surgery may pose a higher risk [14], as well as for the treatment of residual tumors [13,15]. This shift has provided new avenues for managing cases where surgery alone may not be feasible or sufficient. Recent advancements in imaging techniques, including molecular and functional magnetic resonance imaging (MRI), have further improved SRS precision in targeting recurrent lesions [16,17,18].

Despite these advantages, however, challenges remain in the application of SRS for recurrent meningiomas. Notably, the risk of radiation necrosis (RN) may increase significantly in the setting of repeat SRS [19]. For example, the incidence of RN has been shown to be as great as 34.6% following SRS for intracranial recurrent meningioma, imposing dose limitations [20]. Additionally, cumulative radiation exposure raises concern for rare but serious long-term complications, such as radiation-induced meningiomas (RIMs), particularly in younger patients or those undergoing multiple courses of cranial irradiation [21,22]. While RIMs typically develop after long latency periods, prior studies have reported elevated risks with higher total doses and younger age at initial treatment, suggesting that re-irradiation may contribute to lifetime risk in select populations [21,23].

Furthermore, while other CNS tumors such as lower-grade gliomas (LGG) may benefit from systemic agents like everolimus, tyrosine kinase inhibitors, or monoclonal antibodies, recurrent meningiomas lack effective systemic treatment options [24,25]. In addition, the clinical heterogeneity of recurrent meningiomas, influenced by factors such as prior treatments, tumor grade, and molecular profiles, complicates the development of universal SRS plans [26,27]. Recent updates to the WHO Classification of CNS tumors have emphasized the prognostic importance of specific molecular alterations, such as *TERT* promoter mutations and homozygous deletions of *CDKN2A/B* [28,29]. These alterations are associated with more aggressive tumor behavior and poorer clinical outcomes [28,29]. Incorporating molecular data into treatment planning may improve risk stratification and inform the optimal use of SRS in this heterogeneous population.

Given the limited treatment alternatives, complexities, and potential for re-irradiation toxicity, careful patient stratification and individualized radiation planning are crucial for optimizing outcomes in recurrent meningioma. Therefore, a deeper understanding of prognostic factors and treatment parameters in SRS is essential. In this systematic review, we aim to summarize the current evidence for the role of SRS in managing recurrent meningiomas. We further explore individualized treatment planning, optimal SRS dosing strategies, and potential complications.

## 2. Materials and Methods

### 2.1. Search Strategy

A systematic review was conducted in accordance with the Preferred Reporting Items for Systematic Reviews and Meta-Analyses (PRISMA) guidelines [30]. The study protocol was not registered with the Cochrane Database of Systematic Reviews. To identify relevant studies, we queried the PubMed, Scopus, and Web of Science database in January, 2025 using a combination of the following terms: “recurrent” OR “recurrence” AND “meningioma” AND “radiosurgery”. The selection and screening of the articles was conducted through Rayyan (Rayyan Systems Inc., Doha, Qatar).

### 2.2. Eligibility Criteria

Studies were included if they met the following criteria: (1) patients with pathologically confirmed intracranial meningioma who underwent SRS for disease recurrence; and (2) publications in English. Meningiomas of all grades were included. Notably, studies reporting outcomes across multiple tumor types were included, provided that results for recurrent meningiomas treated with SRS were separately reported.

Studies were excluded if they met any of the following criteria: (1) commentaries, reviews, and other articles not containing primary data; (2) case reports (defined as fewer than three cases); or (3) the full text was inaccessible.

### 2.3. Data Extraction and Quality Assessment

Relating to patient characteristics, we collected data on the patient population and the number of treated lesions. For tumor-related factors, we recorded tumor grade at recurrence, gross tumor volume (GTV), and history of prior radiation therapy (RT).

For SRS treatment parameters, we summarized the type of SRS used, median marginal dose, and planning target volume (PTV). Outcome measures included progression-free survival (PFS) and details on local tumor control. Additionally, data on adverse effects were collected. Furthermore, we extracted statistical analyses from studies in which these were included. The quality and risk of bias of each eligible study were assessed using the modified Newcastle–Ottawa Scale (NOS), evaluating selection, comparability, and outcome domains [31].

## 3. Results

### 3.1. Search Results

**Figure 1:**
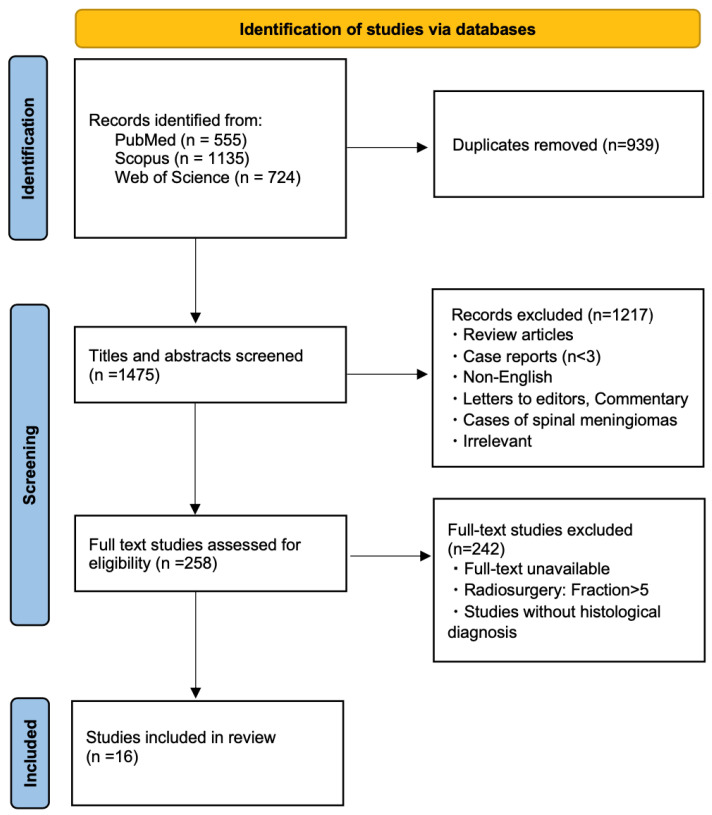
PRISMA flowchart depicting study selection.

As illustrated in the PRISMA diagram (Figure 1), our initial database search retrieved 2414 items, which were reduced to 1475 following removal of duplicates. From these, 1217 studies were excluded based on title and abstract screening, while 242 were removed during full-text assessment.

Accordingly, 16 studies met criteria for inclusion in this review [26,32,33,34,35,36,37,38,39,40,41,42,43,44,45,46]. All included studies were assessed as having a moderate risk of bias using the NOS. Given the moderate heterogeneity across studies, a meta-analysis was not performed in this review.

### 3.2. Stereotactic Radiosurgery for Management of WHO Grade I Recurrent Meningiomas

Table 1 summarizes the five included studies reporting SRS outcomes for WHO Grade I recurrent meningiomas [32,35,38,44,45]. Among these, Kaprealian et al. included cases of recurrence following either surgery alone or RT alone, while the remaining four studies enrolled patients with a history of surgical resection [35]. For tumors that recurred following surgery, reported local control rates ranged from 71% to 100%. Tumors recurring after RT alone demonstrated a local control rate of 62%. Three studies reported the prescribed marginal dose for SRS, with values including a range of 13–20 Gy, a mean of 15.4 Gy, and a median of 13 Gy.

### 3.3. Stereotactic Radiosurgery for Management of WHO Grade II Recurrent Meningiomas

Table 2 summarizes 10 studies that reported outcomes of SRS for WHO Grade II recurrent meningiomas. Kaprealian et al. included cases of recurrence following surgery alone as well as RT alone [35], while Momin et al. examined recurrences after surgery alone and combined RT + surgery [41]. Among tumors with prior surgical resection, local control rates ranged from 40% to 89%. Tumors that recurred after RT alone demonstrated a local control rate of 36%. Reported 3-year PFS rates ranged from 23% to 100%. In the study by Momin et al., the 3-year PFS was 60.7% for tumors treated after surgery alone and 41.0% for those treated after RT + surgery. Four studies reported median marginal dose, with values ranging from 15 Gy to 16 Gy.

### 3.4. Stereotactic Radiosurgery for Management of WHO Grade III Recurrent Meningiomas

Table 3 summarizes three studies that reported outcomes of SRS for WHO Grade III recurrent meningiomas [32,35,39]. Among tumors that had undergone prior surgical resection, reported local control rates were 0%, 79%, and 92% across the respective studies. Tumors that recurred after RT alone showed a local control rate of 31%. Regarding PFS, Mattozo et al. reported a 1-year PFS of 0% [32] and Acker et al. reported a 2-year PFS of 46% [39]. The mean marginal dose reported in one study was 19.3 Gy.

### 3.5. Stereotactic Radiosurgery for Management of High-Grade (Both WHO Grade II and III)/Unknown Grade Recurrent Meningiomas

Outcomes of SRS for high-grade (both WHO Grade II and III) or unknown-grade recurrent meningiomas are summarized in Table 4. Three studies reported combined outcomes for WHO Grade 2 and 3 tumors, with 3-year PFS rates of 48.3% and 57%, and 5-year PFS rates of 33.6% and 48.3% [33,42,46]. Kano et al. reported 5-year PFS rates of 29.4% in patients receiving a marginal dose of <20 Gy and 63.1% in those receiving ≥20 Gy [33].

Hung et al. reported outcomes of SRS for cavernous sinus meningiomas (CSM) recurring after surgery alone, reporting a local control rate of 86% with a median marginal dose of 12 Gy [37].

### 3.6. Stereotactic Radiosurgery for Recurrent Meningiomas After Surgery Alone

Table 5 summarizes five studies that reported outcomes of SRS for recurrent meningiomas treated after surgical resection alone [35,37,41,44,45]. Reported local control rates ranged from 79% to 100%. Three studies provided 5-year PFS rates ranging from 40.4% to 91%. Momin et al. additionally reported a 3-year PFS rate of 60.7%. The reported median marginal dose ranged from 12 Gy to 16 Gy.

### 3.7. Stereotactic Radiosurgery for Recurrent Meningiomas After RT +/− Surgery

Table 6 summarizes two studies that reported outcomes of SRS for recurrent meningiomas previously treated with RT with or without surgical resection [35,41]. Momin et al. reported a 3-year PFS rate of 41.0% for tumors that recurred following RT and surgery [41]. Kaprealian et al. reported outcomes for tumors that recurred after RT alone, with a 5-year PFS of 26% and local control rates ranging from 31% to 62% depending on WHO grade [35]. The median marginal doses reported in the two studies were 15 Gy and 16 Gy, respectively.

Although several additional studies included patients with a history of both surgery and RT, they reported outcomes only for the overall cohort and did not provide stratified results for patients with prior RT. Therefore, only two studies presented outcome data specifically for recurrent tumors after RT.

### 3.8. Treatment-Related Toxicity of Stereotactic Radiosurgery for Recurrent Meningioma

Treatment-related toxicity associated with SRS for recurrent meningiomas was reported in eight studies (Table 7). Kaprealian et al. assessed adverse radiation effects (AREs) in patients with recurrence after surgery alone and after RT alone, with 1- and 2-year probabilities of 5% and 5% in the former group, and 15% and 30% in the latter group, respectively [35]. Reported rates of SRS-related toxicity ranged from 3.7% to 37%. Radiation necrosis was reported in three studies [26,44,46].

### 3.9. Statistical Analysis Results for Studies Including Only Salvage SRS

Table 8 summarizes the results of univariable and multivariable analyses from five studies that included patients who underwent salvage SRS for recurrent meningioma [26,33,34,39,46]. All five studies conducted univariable analyses, while Acker et al. and Gallitto et al. also performed multivariable analyses [39,46]. The included recurrent meningiomas were all WHO Grade II or III.

Kano et al., Valery et al., and Gallitto et al. reported that radiation dose significantly influenced outcomes in univariable analysis [26,33,46]. Kano et al. reported that a marginal dose of <20 Gy was a predictor of worse PFS (*p* < 0.05) [33]. Valery et al. reported that treatment with a minimum dose of ≤12 Gy was associated with increased local relapses (*p* = 0.04) [26]. Gallitto et al. identified a median marginal radiation dose as a predictor of worse PFS, with a hazard ratio (HR) of 1.09 (95%CI 1.01–1.18), *p* = 0.024 [46]. In contrast, Aboukais et al. and Acker et al. did not find radiation dose to be a significant factor in univariable analysis [34,39]. However, in multivariable analysis, Acker et al. identified mean EQD_2_ as a significant risk factor for local recurrence in Grade II meningiomas (HR 1.210 [1.070–1.367], *p* = 0.002) [39].

Several studies also suggested that prior RT before SRS influenced outcomes. Aboukais et al. found that postoperative RT was a significant factor affecting regional tumor control in univariable analysis (*p* = 0.0254) [34]. Gallitto et al. reported that prior RT history was a predictor of worse PFS in univariable analysis (HR 1.85 [1.10–3.12], *p* = 0.02) and remained significant in multivariable analysis (HR 2.69 [1.23–5.86], *p* = 0.013) [46]. Histological grade also played a role in prognosis. Gallitto et al. found that Grade 3 histology was a significant predictor of worse PFS in both univariable (HR 11.40 [3.95–33.0], *p* < 0.001) and multivariable analysis (HR 6.80 [1.61–28.6], *p* = 0.009) [46].

Age was another significant prognostic factor in two studies. Aboukais et al. and Acker et al. identified age as a significant risk factor in univariable analysis, which was *p* = 0.0496 and *p* = 0.002, respectively [34,39]. Furthermore, Acker et al. reported that age remained a significant risk factor for local recurrence in Grade II meningiomas in multivariable analysis (*p* = 0.002) [39]. Regarding tumor volume, Aboukais et al. found in univariable analysis that it was a significant factor affecting local tumor control (*p* = 0.0445), although it was not examined in multivariable analysis [34].

## 4. Discussion

### 4.1. Overall Effectiveness of SRS in Recurrent Meningiomas by Tumor Grade

This review highlights the overall effectiveness of SRS as a salvage treatment for recurrent meningiomas, with outcomes varying by WHO grade. WHO Grade I recurrent meningiomas achieved consistently high local control rates, ranging from 62% to 100%. These findings suggest that low-grade tumors remain radiosensitive even in the recurrent setting, particularly when treated after initial surgical resection. These findings are consistent with prior studies such as Kondziolka et al., which reported 5-year local control rates over 90% in benign meningiomas treated with SRS [12].

While these findings support the efficacy of SRS in recurrent WHO Grade I meningiomas, they appear more favorable compared to those reported in the RANO review by Kaley et al., which evaluated systemic therapies in patients with surgery- and radiation-refractory meningiomas [47]. In that analysis, the 6-month PFS for WHO Grade I tumors was only 29%, highlighting the limited efficacy of medical treatments in this heavily pretreated population [47]. Although direct comparisons are limited by differences in treatment context and patient selection, this difference underscores the potential value of SRS as a favorable salvage option in select cases of recurrent Grade I meningiomas.

For WHO Grade II meningiomas, LC rates were more variable, ranging from 40% to 89%, and 3-year PFS rates ranged from 23% to 100%. While some patients experienced durable control, others showed early progression, indicating a need for better risk stratification. In contrast, outcomes for WHO Grade III meningiomas were generally poor, with one study reporting a 2-year PFS rate below 50% [39] and another reporting a 1-year PFS rate of 0% [32]. These results align with previous studies highlighting the aggressive nature of high-grade meningiomas [48].

### 4.2. Overall Effectiveness of SRS in Recurrent Meningiomas by Prior Treatment

In addition to tumor grade, prior treatment history significantly influenced SRS outcomes. Patients whose tumors recurred after surgery alone tended to achieve favorable outcomes with SRS, with reported favorable LC rates ranging from 79% to 100%. On the other hand, tumors that recurred after prior RT with or without surgery demonstrated significantly lower control rates. LC in these cases ranged from 31% to 62%, and 5-year PFS was as low as 26%.

Two studies, by Momin et al. and Kaprealian et al., provided comparative data across treatment histories and tumor grades [35,41]. In Momin et al., WHO Grade II patients who received SRS after surgery alone had a 3-year PFS of 60.7% and a 5-year PFS of 40.4%, compared to a 3-year PFS of 41.0% in those with prior RT, despite similar marginal doses. Kaprealian et al. similarly demonstrated that prior RT was associated with significantly reduced local control across all grades. In the surgery-alone cohort, 5-year LC was 87% (Grade I), 79% (Grade II), and 92% (Grade III). In contrast, the RT-treated cohort showed lower LC of 62% (Grade I), 36% (Grade II), and 31% (Grade III), despite comparable doses.

These findings indicate that prior RT not only diminishes the efficacy of SRS but does so in a grade-dependent manner, with WHO Grade II and III tumors showing the greatest reductions in local control rates, likely reflecting intrinsic radioresistance and more aggressive tumor biology. Accordingly, both histopathological grade and treatment history emerge as key prognostic factors in guiding SRS for recurrent meningiomas.

Recent molecular evidence may further explain these clinical observations. Wang et al. reported that RT-resistant meningiomas demonstrate a proliferative transcriptional profile enriched for cell cycle progression and DNA damage repair, with concurrent suppression of apoptotic pathways [49]. Furthermore, a large-scale genomic analysis by Patel et al. identified three distinct molecular subtypes of meningiomas predictive of recurrence, independent of WHO grade, underscoring the biological heterogeneity not captured by conventional histopathological classification [50]. These findings underscore the importance of incorporating both tumor biology and prior radiation history into personalized SRS planning to improve patient selection and optimize therapeutic outcomes.

### 4.3. Prognostic Factors for SRS Outcomes

Several prognostic factors for improved outcomes were identified. Prior radiation exposure emerged as a major prognostic factor negatively impacting SRS outcomes, particularly in WHO Grade II and III tumors. Across multiple studies, patients with previously irradiated tumors experienced significantly lower local control and PFS than those undergoing surgery alone, suggesting acquired radioresistance and more aggressive tumor biology [34,35,41,46]. These studies consistently showed that previously irradiated patients had lower PFS and local control compared to those without prior RT [51].

In addition, radiation dose influenced efficacy. Across studies, a higher marginal dose was consistently associated with better tumor control, particularly in Grade II/III tumors. For instance, PFS was significantly worse with doses ≤12 Gy, while doses ≥20 Gy improved outcomes [26,33,46]. These findings are in line with prior dose-response studies emphasizing the importance of adequate dosing for durable tumor control [52,53]. Sethi et al. recommended escalating the dose to 16–20 Gy in high-grade meningiomas when feasible [52], and Wang et al. suggested marginal doses >13 Gy along with maximal safe resection to enhance local control [53]. Other clinical factors such as histological grade, older age, and larger tumor volume were also associated with worse outcomes in several studies [34,43,46]. These findings align with Ferraro et al., who reported that both WHO Grade III diagnosis and larger treated tumor volume independently predicted worse survival, regardless of whether SRS was used as adjuvant or salvage therapy [54].

Taken together, these findings highlight the importance of individualized SRS planning that carefully considers tumor grade, prescribed dose, tumor volume, patient age, and, most importantly, a history of prior radiation therapy.

Despite these insights, none of the 16 studies included molecular or epigenetic tumor profiling, such as TERT promoter mutations, CDKN2A/B deletions, or methylation subclasses, which are increasingly shown to correlate with prognosis and RT responsiveness. Recent studies highlight that molecular profiling may better predict recurrence and radiosensitivity than WHO grade alone. Sahm et al. demonstrated that methylation-based classification outperforms histology in prognosticating recurrence risk, especially in Grade I–II tumors [55]. Nassiri et al. proposed a four-subtype molecular framework correlating with clinical outcomes [56,57], and Wang et al., incorporating the RTOG-0539 cohort, showed that these subtypes more accurately predict RT response, particularly noting that the proliferative subtype derives minimal benefit from radiation [49]. These findings suggest that integration of molecular classification into clinical and research settings will be essential to improve patient selection for radiosurgery, guide adjuvant therapy decisions, and enable the design of molecularly informed clinical trials.

### 4.4. Salvage Strategies After SRS Failure

Despite the favorable local control rates achieved with SRS in selected patients, tumor recurrence remains a clinical challenge. Salvage strategies following SRS failure vary depending on the location of recurrence, tumor size, and prior treatments. Aboukais et al. treated most regional or in-field recurrences with repeat SRS or surgery, without treatment-related morbidity or technical difficulties, suggesting that both repeat SRS and resection are feasible salvage options [34]. Valery et al. also reported repeat Gamma Knife in 14 cases, with additional surgery, fractionated RT, or chemotherapy in select patients [26]. In a larger cohort, Gallitto et al. reported that salvage therapies for recurrence after SRS included fractionated RT (47%), surgery (38%), and repeat SRS (6%) [46].

Collectively, these studies highlight that multiple salvage approaches, including repeat SRS, surgical resection, and fractionated radiotherapy, are feasible and variably employed depending on the clinical context. However, the lack of standardized protocols and limited prospective data complicate decision-making in this setting. Given the predominance of local and marginal failures, especially in Grade II and III tumors, further investigation into optimal dose parameters, margin definitions, and patient selection criteria is warranted.

### 4.5. Toxicity, Radiation Necrosis, and Predictive Factors

In addition to efficacy, treatment-related complications are a critical consideration when evaluating the use of SRS for recurrent meningiomas. Reported adverse event rates ranged from 3.7% to 37% across studies [34,39], with seizures and visual disturbances being common toxicities among the studies. One consistent factor associated with increased toxicity is prior RT. Kaprealian et al. reported that the 2-year probability of adverse radiation effects (AREs) reached 30% in patients who had undergone previous RT, compared to only 5% in patients treated after surgery alone [35]. Similarly, Acker et al. and Valery et al. reported high rates of treatment-related toxicities (37% and 22%, respectively), with a significant proportion of affected patients having a history of RT [26,39]. These findings strongly suggest that cumulative radiation exposure sensitizes surrounding tissues, potentially increasing vulnerability to radiation-induced damage.

RN is another well-recognized complication that can arise after initial or salvage SRS [58], and understanding predictive factors is crucial when considering retreatment. In the multi-institutional study led by Gallitto et al., the incidence of grade II or higher radiation necrosis was 3.0%, indicating that clinically significant RN may not be frequent but still warrants consideration [46]. One major predictor is the volume of normal brain receiving high dose, typically quantified as 12-Gy radiosurgical volume (V12) in single-fraction SRS. Korytko et al. found symptomatic RN exceeded 50% when V12 > 10 cc [59]. However, Parikh et al. reported 6.9% symptomatic toxicity despite a median V12 of 11.3 cc, underscoring that V12 alone may not fully capture RN risk in complex cases [60]. Further supporting this, Milano et al., through an American Association of Physicists in Medicine (AAPM) pooled analysis of 51 studies, demonstrated that V12 values of 5, 10, and >15 cm^3^ were associated with approximate risks of symptomatic radionecrosis of 10%, 15%, and 20%, respectively [61]. Importantly, they also identified that in fractionated SRS (fSRS), keeping the V20 (for 3 fractions) or V24 (for 5 fractions) below 20 cm^3^ was associated with <10% risk of necrosis or edema and <4% risk of radionecrosis requiring resection [61]. These findings emphasize the importance of individualized dose planning and suggest that fSRS may offer a safer alternative to single-fraction SRS in cases requiring larger target volumes or re-irradiation.

Beyond standard dose-volume thresholds such as V12 or V20, multiple studies have demonstrated that tumor size and prescription dose have also been shown to independently affect RN risk. Kerschbaumer et al. revealed that both increased tumor diameter and higher prescription dose were significant independent predictors of RN, with risk rising by over 180% between 14 Gy and 20 Gy, and each 1 mm increase in tumor diameter associated with elevated RN risk [20]. Similarly, Doré et al. reported that in the postoperative SRS setting for brain metastases, both the preoperative tumor size and V21 significantly predicted the development of RN [62]. These findings highlight that while dosimetric constraints remain essential, individualized planning must also incorporate tumor volume and dose intensity, especially in cases with large resection cavities or bulky disease, where the risk of RN may outweigh the potential benefit of aggressive local control.

Taken together, these studies highlight the importance of careful treatment planning that considers prior RT, target volume, and precise dose constraints. When these parameters are respected, salvage SRS can remain a safe and effective option for selected patients with recurrent meningiomas, offering disease control with an acceptable toxicity profile.

### 4.6. Limitations and Future Directions

This study has several limitations. Most studies were retrospective in design and had a moderate risk of bias as assessed by NOS. The heterogeneity in SRS platforms, dose protocols, tumor characteristics, and outcome definitions precluded meta-analysis and limited direct comparisons. Moreover, none incorporated molecular or epigenetic profiling, despite growing evidence that such markers affect radiosensitivity and prognosis.

Future prospective multicenter studies with standardized protocols are needed not only to define optimal SRS dose thresholds and clarify the role of re-irradiation in previously irradiated patients, but also to integrate molecular profiling into patient selection, prognostication, and treatment planning.

In parallel, the combination of SRS with immunotherapy is emerging as a promising strategy for patients with recurrent high-grade and radiation-relapsed meningiomas. A recent phase I/II clinical trial (NCT03604978) assessing SRS with nivolumab showed favorable tolerability, supporting further exploration of this approach [63].

## 5. Conclusions

This systematic review suggests that SRS may be a reasonable salvage option for carefully selected patients with recurrent meningiomas, especially those who recur after surgery alone. In contrast, outcomes were significantly worse for tumors that recurred after prior radiotherapy, particularly in high-grade tumors. The findings emphasize the prognostic importance of both histopathological grade and treatment history. However, the current literature lacks molecular or genetic tumor characteristics. Future prospective studies integrating molecular profiling are needed to improve risk stratification and guide individualized SRS-based treatment strategies.

## Figures and Tables

**Table 1:** Summary of included studies of stereotactic radiosurgery for management of Grade I recurrent meningiomas.

Study	#Patients/ #Tumors	Prior Treatment	Median GTV/PTV (cm^3^)	Salvage Treatment	Median Marginal Dose (Gy)	PFS (%)	Local Control (%)	Local Control Definition
Rock, 2024 [45]	22/-	Surgery: 100% RT: 0%	-	LINAC	Range: 13–20	5-yr PFS: 91	100	No tumor progression (>20% tumor volume increase)
Schmutzer, 2023 [44]	71/71	Surgery: 100% RT: 0%	Mean: 7.0/-	CyberKnife	Mean: 15.4	-	91.2	Tumor shrinkage and/or no size change
Park, 2019 [38]	34/-	Surgery: 100% RT: 6%	5.9/-	Gamma Knife	13	5 yr PFS: 85	71	No tumor progression (>25% tumor volume increase)
Kaprealian, 2016 [35]	-/96	Surgery: 80% RT: 20%	-	Gamma Knife	-	-	Prior surgery: 87 Prior RT: 62 (5-year)	Freedom from progression (>20% tumor volume increase)
Mattozo, 2007 [32]	-/3	Surgery: 100% RT: -	-	LINAC	-	3 yr PFS: 100	100	No increase in lesion size

Gy: gray, yr: year, LINAC: linear accelerator, GTV: gross tumor volume, PTV: planning target volume, RT: radiation therapy, PFS: progression-free survival.

**Table 2:** Summary of included studies of stereotactic radiosurgery for management of Grade II recurrent meningiomas.

Study	#Patients/ #Tumors	Prior Treatment	Median GTV/PTV (cm^3^)	Salvage Treatment	Median Marginal Dose (Gy)	PFS (%)	Local Control (%)	Local Control Definition
Mattozo, 2007 [32]	-/19	Surgery: 100% RT: -	-	LINAC	-	3-yr PFS: 100	89	No increase in lesion size
Aboukais, 2015 [34]	27/27	Surgery: 100% RT: 30%	-/5.4	Gamma Knife	Mean: 15.2 (Prior surgery alone: Mean 14.9, Prior RT: Mean 15.9)	-	75, 52, 40 (1-, 2-, 3-year)	Size reduction or stability
Kaprealian, 2016 [35]	-/48	Surgery: 44% RT: 56%	-	Gamma Knife	-	-	Prior surgery: 79 Prior RT: 36 (5-year)	Freedom from progression (>20% tumor volume increase)
Valery, 2016 [26]	18/58	Surgery: 100% RT: 39%	2.5/-	Gamma Knife	Range: 14–16	3-yr PFS: 23	89, 71 (1-, 3-year)	No tumor progression (>20% tumor volume increase)
Chen, 2018 [36]	24/-	Surgery: 100% RT: 29%	-	-	15	-	90, 66, 44 (1-, 2-, 3-year)	No local recurrence
Acker, 2019 [39]	27/105	Surgery: 100% RT: 48%	-/1.55	CyberKnife	Mean: 23.1	73, 59 (3-, 5-year)	84	No lesion progression
Hasegawa, 2021 [40]	17: Early salvage (7–18 months); 33: Late salvage (>18 months)/-	Surgery: 100% RT: -	-	Gamma Knife	16	Early salvage: 33, 0 (3-, 5-year) Late salvage: 61, 48 (3-, 5-year)	46 (3-year)	No tumor progression (>20% tumor volume increase)
Momin, 2021 [41]	-/51	Surgery: 100% RT: 67%	Mean: 2.2/-	Gamma Knife	Prior surgery: 16, Prior RT: 15	Prior surgery alone: 60.7, 40.4 (3-, 5-year), Prior RT: 41.0 (3-year)	-	-
Marchetti, 2022 [43]	16/-	Surgery: 100% RT: -	-	CyberKnife	28 Gy in 4 fractions or 24 Gy in 4 fractions	-	75	No tumor progression
Gallitto, 2024 [46]	102/102	-	-	Gamma Knife or LINAC	16	3-yr PFS: 64	-	-

Gy: gray, yr: year, LINAC: linear accelerator, GTV: gross tumor volume, PTV: planning target volume, RT: radiation therapy, PFS: progression-free survival.

**Table 3:** Summary of included studies of stereotactic radiosurgery for management of Grade III recurrent meningiomas.

Study	#Patients/ #Tumors	Prior Treatment	Median GTV/PTV (cm^3^)	Salvage Treatment	Median Marginal Dose (Gy)	PFS (%)	Local Control (%)	Local Control Definition
Mattozo, 2007 [32]	-/5	Surgery: 100% RT: -	-	LINAC	-	1-yr PFS: 0	0	No increase in lesion size
Kaprealian, 2016 [35]	-/76	Surgery: 16% RT: 84%	-	Gamma Knife	-	-	Prior surgery: 92 Prior RT: 31 (5-year)	Freedom from progression (>20% tumor volume increase)
Acker, 2019 [39]	8/22	Surgery: 100% RT: 50%	2.38/-	CyberKnife	Mean: 19.3	2-yr PFS: 46	79	No lesion progression

Gy: gray, yr: year, LINAC: linear accelerator, GTV: gross tumor volume, PTV: planning target volume, RT: radiation therapy, PFS: progression-free survival.

**Table 4:** Summary of included studies of stereotactic radiosurgery for management of high-grade (both Grade II and III)/unknown grade recurrent meningiomas.

Study	#Patients/ #Tumors	WHO Grade	Prior Treatment	Median GTV/PTV (cm^3^)	Salvage Treatment	Median Marginal Dose (Gy)	PFS (%)	Local Control (%)	Local Control Definition
Kano, 2007 [33]	12/30	G2 (*n* = 10); G3 (*n* = 2)	Surgery: 100% RT: 33%	-/2.87	LINAC	19	3-, 5-yr PFS: 48.3 [5-yr PFS: 29.4 (<20 Gy), 63.1 (20 Gy)]	57	No in-field recurrence
Shepard, 2021 [42]	141/-	G2, G3	Surgery: 100% RT: -	-	Gamma Knife	14.8	66.6, 33.6 (2-, 5-year)	-	-
Gallitto, 2024 [46]	34/-	G2 (*n* = 102); G3 (*n* = 6)	Surgery: 98% RT: 19%	2.80/-	Gamma Knife or LINAC	16	3-yr PFS: 57	-	-
Hung, 2019 [37]	37/-	- (CSM)	Surgery: 100% RT: 0%	-	Gamma Knife	12	-	86	No tumor progression (defined as volume >110% of original)

Gy: gray, yr: year, LINAC: linear accelerator, GTV: gross tumor volume, PTV: planning target volume, RT: radiation therapy, PFS: progression-free survival, CSM: cavernous sinus meningioma, G: grade.

**Table 5:** Summary of selected studies of stereotactic radiosurgery for recurrent meningiomas after surgery alone.

Prior Resection Patients (%)	Prior RT Patients (%)	Study	WHO Grade	Median Marginal Dose (Gy)	PFS (%)	Local Control (%)	Local Control Definition
100	0	Schmutzer, 2023 [44]	G1	Mean: 15.4	-	91.2	tumor shrinkage and/or no size change
		Rock, 2024 [45]	G1	Range: 13–20	5-yr PFS: 91	100	No tumor progression (>20% tumor volume increase)
		Hung, 2019 [37]	- (CSM)	12 (11–21)	-	86	No tumor progression (defined as volume >110% of original)
		Momin, 2021 [41]	G2	16 (12–18)	60.7, 40.4 (3-, 5-year)	-	-
		Kaprealian, 2016 [35]	G1, G2, G3	15 (12–20)	5-yr PFS: 82	5-year: G1 87, G2 79, G3 92	Freedom from progression (>20% tumor volume increase)

Gy: gray, yr: year, RT: radiation therapy, PFS: progression-free survival, CSM: cavernous sinus meningioma, G: grade.

**Table 6:** Summary of selected studies of stereotactic radiosurgery for recurrent meningiomas after RT +/− surgery.

Prior Resection Patients (%)	Prior RT Patients (%)	Study	WHO Grade	Median Marginal Dose (Gy)	PFS (%)	Local Control (%)	Local Control Definition
100	100	Momin, 2021 [41]	G2	15 (13–20)	3-yr PFS: 41.0	-	-
0	100	Kaprealian, 2016 [35]	G1, G2, G3	16 (12–19)	5-yr PFS: 26	5-year: G1 62, G2 36, G3 31	Freedom from progression (>20% tumor volume increase)

Gy: gray, yr: year, RT: radiation therapy, PFS: progression-free survival, G: grade.

**Table 7:** Summary of studies that reported side effects of SRS treatment.

Prior Resection Patients (%)	Prior RT Patients (%)	Study	Median Marginal Dose (Gy)	SRS Treatment-Related Toxicity
100	0	Schmutzer, 2023 [44]	Mean: 15.4	3.6% (headache), 2.9% (perifocal edema), 2.2% (seizures), 2.2% (vertigo), 0.7% (radiation necrosis)
100	0	Momin, 2021 [41]	16 (12–18)	29.4% (CTCAE grade ^a^ ≥ I adverse events. Most common were alopecia, dermatitis, fatigue, and headache. No radiation necrosis.)
100	0	Kaprealian, 2016 [35]	15 (12–20)	5%, 5% (1-, 2-year probability of ARE)
98	19	Gallitto, 2024 [46]	16	3% (radiation necrosis), 7% (cognitive disturbance), 4% (new-onset seizures)
100	30	Aboukais, 2015 [34]	Mean: 15.2	3.7% (transient hemiparesis)
100	33	Kano, 2007 [33]	19 (12–20)	17% (asymptomatic perifocal edema from radiation-induced angiopathy)
100	39	Valery, 2016 [26]	Range: 14–16	22% (2 radiation necrosis treated by corticosteroids, 1 spontaneous hemorrhage, 1 recurrent seizures)
100	49	Acker, 2019 [39]	16 (15–18)	37% (6 focal seizures, 2 mild visual deterioration, 2 dysesthesia, 1 fatigue, 1 headache, 1 fine motor skill disturbance)
0	100	Kaprealian, 2016 [35]	16 (12–19)	15%, 30% (1-, 2-year probability of ARE)

Gy: gray, RT: radiation therapy, PFS: progression-free survival, CSM: cavernous sinus meningioma, G: grade, SRS: stereotactic radiosurgery, ARE: adverse radiation effects. ^a^ Radiation toxicities were collected and graded according to the Common Terminology Criteria for Adverse Events (CTCAE) version 5.0 (http://ctep.cancer.gov).

**Table 8:** Summary of reported outcome-predicting factors.

Study	WHO Grade	Univariable Analysis Significant (HR (95%CI))	Univariable Analysis Not Significant (HR (95%CI))	Multivariable Analysis Significant (HR (95%CI))	Multivariable Analysis Not Significant (HR (95%CI))
Kano, 2007 [33]	G2 (n = 10); G3 (n = 2)	Predictors of worse PFS: Marginal radiation dose (<20 Gy) (*p* < 0.05)	Sex, age, tumor location, target volume, tumor grade	-	-
Aboukais, 2015 [34]	G2	Factors that may have affected LC: Age (*p* = 0.0496), target volume (*p* = 0.0445) Factors that may have affected regional control: Sex (*p* = 0.0333), no. of resections (*p* = 0.0310), postop RT (*p* = 0.0254)	Regarding LC: Sex, location of recurrence, Simpson grade, no. of resections, postop RT, delay between surgery and SRS, radiation dose Regarding regional control: Age, location of recurrence, Simpson grade, delay between surgery and SRS, radiation dose, target volume	-	-
Valery, 2016 [26]	G2	Factors for more local relapses: treated with a minimum dose of ≤12 Gy (*p* = 0.04) Factors for improved marginal control: Tumor growth rate (*p* = 0.002) Factors for worse PFS: delay between first surgery and GKRS (*p* = 0.03)	Regarding LC: Tumor growth rate, tumor volume	-	-
Acker, 2019 [39]	G2 (n = 27); G3 (n = 8)	Risk factors in G2 meningioma for local recurrence: Age 1.133 (1.046–1.227), *p* = 0.002	Gender, planning target volume, prescribed dose, minimal dose, mean dose, maximal dose, dose mean EQD_2_, coverage	Risk factors in G2 meningioma for local recurrence: Age 1.104 (1.038–1.175), *p* = 0.002 Dose mean EQD_2_ 1.210 (1.070–1.367), *p* = 0.002	Gender
Gallitto, 2024 [46]	G2 (n = 102); G3 (n = 6)	Predictors of worse PFS: G3 histology 11.40 (3.95–33.0), *p* < 0.001 Median marginal radiation dose (Gy) 1.09 (1.01–1.18), *p* = 0.024 History of prior RT 1.85 (1.10–3.12), *p* = 0.02	Age, male gender, tumor volume, maximum point dose	Predictors of worse PFS: G3 histology 6.80 (1.61–28.6), *p* = 0.009 History of prior RT 2.69 (1.23–5.86), *p* = 0.013 Male gender 3.48 (1.47–8.26), *p* = 0.005	Age, tumor volume, median marginal radiation dose, maximum point dose

EQD_2_: equivalent dose, LC: local control, G: grade, GKRS: gamma knife radiosurgery, Gy: gray, HR: hazard ratio, NR: not reported, PFS: progression-free survival, RT: radiation therapy, SRS: stereotactic radiosurgery.
